# Age at cytomegalovirus, Epstein Barr virus and varicella zoster virus infection and risk of atopy: The Born in Bradford cohort, UK

**DOI:** 10.1111/pai.13093

**Published:** 2019-06-28

**Authors:** Lucy Pembrey, Dagmar Waiblinger, Paul Griffiths, John Wright

**Affiliations:** ^1^ Department of Medical Statistics London School of Hygiene and Tropical Medicine London UK; ^2^ Bradford Institute for Health Research Bradford UK; ^3^ Centre for Virology University College London Medical School London UK

**Keywords:** age at infection, atopy, birth cohort, cytomegalovirus, Epstein‐Barr virus

## Abstract

**Background:**

The prevalence of allergic diseases has increased in recent decades, but the causes remain unclear. Changes in the epidemiology of childhood infections could have contributed, but the current evidence is inconclusive. This study aims to investigate whether age at cytomegalovirus (CMV), Epstein‐Barr virus (EBV) or varicella zoster virus (VZV) infection is associated with the development of atopy.

**Methods:**

A total of 2559 children were enrolled in the Born in Bradford Allergy and Infection Study. Serum samples collected at 12 and 24 months were tested for CMV‐IgG, EBV‐IgG and VZV‐IgG for 1000 children to establish age at infection. Skin prick testing (SPT) was conducted at age 4 years.

**Results:**

Serology and SPT results were available for 740 children. Of these, 135 (18%) were atopic. In girls, there was a strong association of CMV infection in the second year with increased odds of atopy (adjusted OR 4.38, 95% CI 1.87‐10.29) but this was not observed in boys. Age at EBV or VZV infection was not associated with risk of atopy in unadjusted analysis, but there was effect modification by sex; girls infected with VZV in the second year of life had increased odds of atopy (adjusted OR 2.85, 95% CI 1.29‐6.30).

**Conclusions:**

Our results highlight potential sex‐specific effects of age at CMV infection and age at VZV infection on risk of atopy, which provide insight into the mechanisms involved in the development of atopy.

**Key Message**

We present skin prick test data from a large population‐based cohort of children and show an increased odds of atopy among girls infected with cytomegalovirus in the second year of life, compared to those infected in the first year or remaining uninfected at two years. Our results demonstrate the importance of differentiating between infection in the first and second years when investigating associations between common childhood infections and risk of atopy. We highlight potential sex‐specific effects of age at CMV infection on risk of atopy, which may provide insight into the mechanisms involved in the development of atopy.

## 1 INTRODUCTION

The hygiene hypothesis proposes that the dramatic rise in prevalence of eczema, hay fever and allergic asthma in recent decades is due to a reduction in childhood infections.^1^, ^2^ However, many studies have investigated proxy measures for childhood infections such as birth order and childcare^3^, ^4^ or have focussed on symptomatic, clinically diagnosed infections.^5^, ^6^, ^7^

Cytomegalovirus (CMV), Epstein‐Barr virus (EBV) and varicella zoster virus (VZV) are persistent herpesviruses commonly acquired in childhood. CMV and EBV are usually asymptomatic, and VZV causes chickenpox. There is good evidence that CMV and EBV affect the developing immune system.^8^, ^9^ CMV impacts on T‐cell^10^ and NK cell^11^, ^12^ differentiation, and CMV seropositivity is associated with inflammation, atherosclerosis and immunosenescence.^13^, ^14^ EBV is associated with immune disorders such as multiple sclerosis and lymphoma.^15^, ^16^ The features of CMV and EBV in particular indicate that changes in the epidemiology of these infections could have contributed to the increases in eczema, hay fever and allergic asthma.

In a Swedish study, no association was found between CMV or EBV seropositivity at age 4 and asthma, hay fever or eczema, although CMV‐positive/EBV‐negative children were more likely to have specific IgE to common allergens than children seronegative for both infections.^17^, ^18^ In another study, children who were EBV seropositive at 24 months were less likely to be IgE‐sensitized at this age compared to EBV‐seronegative children and this association was enhanced among children co‐infected with CMV.^19^ Another analysis by the same group showed that children infected with EBV before 2 years were less likely to be IgE‐sensitized at age 2 and 5 years while those infected after 2 years were more likely to be IgE‐sensitized at 5 years.^20^

There was no association between CMV, EBV or VZV infection at age 12 months and eczema, asthma, hay fever, total serum IgE levels or allergic sensitization at age 7 in a German study.^21^ In a UK case‐control study, there was no association between EBV or VZV infection and eczema in 1‐ to 4‐year‐olds.^22^ Other studies have examined the association between seropositivity to CMV, EBV, VZV and other infections and risk of atopy or eczema, hay fever, or asthma, but the combined evidence is inconclusive.^23^, ^24^, ^25^ In some, the findings were limited by a lack of statistical power, but in most, infection status was measured at only one time point, often at age 4 or older, so it was not possible to pinpoint age at infection more precisely. As herpesvirus infections are common, it is unlikely that infection per se is associated with risk of disease, but alteration in risk could result from infection at a critical period in immune development. Measuring infection status at more than one age in the first years of life is therefore important. In the Born in Bradford Allergy and Infection Study, children were tested for CMV‐, EBV‐ and VZV‐IgG at 12 and 24 months. We have previously reported earlier infection in Pakistani compared to white British children.^26^ The aim of this analysis is to investigate whether earlier CMV and/or EBV infection protect against the development of atopy.

## 2 METHODS

The Allergy and Infection Study (ALL IN) is a sub‐study of the Born in Bradford birth cohort (BiB), described elsewhere.^27^ Children were eligible to participate if they were enrolled in BiB with a maternal baseline questionnaire available and were born on or after 1 March 2008. Parents were invited to participate in ALL IN just before the child's first birthday. If they agreed, a home or clinic visit was arranged and informed consent was obtained. A blood sample was taken, and a questionnaire was completed at 12 months and again at 24 months of age. Serum aliquots were stored at −80°C. The questionnaires included detailed information on breastfeeding and childcare, and the standard International Study of Asthma and Allergies in Childhood (ISAAC) questions on potential risk factors for atopy (pets, damp, mould, fuel for cooking and heating, type of windows in the child's bedroom, type of flooring, type of pillow and bedding for the child).^28^

Of the 2559 children enrolled in ALL IN, serum samples were tested for CMV‐IgG, EBV‐IgG and VZV‐IgG for 1000 children to establish age at infection; infected by 12 months; infected between 12 and 24 months; or uninfected at 24 months.^26^ Samples with concentration values of ≥6.0 AU/mL were considered as positive for CMV‐IgG, and VCA IgG concentrations of ≥20 U/mL were considered as positive for EBV‐IgG. For VZV, samples containing 160 mIU/mL were considered positive, those between 140 and 160 were equivocal and those less than 140 mIU/mL were deemed negative.

When the children were 4 years old, the parents were re‐contacted and invited for a visit including a questionnaire and skin prick test (SPT), as part of the MeDALL (Mechanisms of the Development of Allergy) study.^29^, ^30^ The SPT was performed according to a standard protocol using the following allergens: cat hair, dog hair, grass mix, house dust mites *Dermatophagoides pteronyssinus* and *Dermatophagoides farinae* plus a positive control (histamine 1%) and a negative saline control (Allergopharma kits supplied by Diagenics Ltd). The mean wheal diameter was recorded for each allergen (and the diameter of any wheal from the negative control subtracted). A wheal diameter of ≥3 mm was considered a positive reaction. Children with a positive reaction to at least one allergen were defined as atopic.

The community research staff were trained in SPT, with quality control procedures to assess intra‐observer variability; all staff had to perform two monthly quality checks and achieve a coefficient of variation of less than 20 in their SPT series.

Parents were given the results of their child's SPT with written guidance on allergen avoidance for those with positive tests.

This study had ethical approval from the London School of Hygiene & Tropical Medicine ethics committee (refs: 5320 and 6249) and the Bradford Research Ethics committee (refs: 08/H1302/21 and 12/YH/0252).

### 2.1 Statistical analysis

Data analysis was conducted using Stata version 14.^31^ Frequency distributions for key variables described the children and their mothers. Cross‐tabulations showed associations between these variables and atopic status. Age at CMV, EBV and VZV infection were the key exposures of interest. The outcome for this analysis was measured at age 4 years only and in all children at the same age. Our data therefore provide an estimate of prevalence, not risk, of atopy, so it is appropriate to present prevalence odds ratios from logistic regression analysis. If univariable analysis demonstrated an association with atopy, the relevant variable was included as a confounder in the multiple logistic regression analysis. The findings for age at CMV infection were compared between the univariable and multiple logistic regression analyses, and no evidence of multicollinearity was found, so the multiple regression analysis was accepted as the final model. Birth order and duration of breastfeeding were included in the CMV model a priori as these are strongly associated with age at CMV infection^26^ and associated with atopy in other studies.^32^, ^33^, ^34^ Effect modification by sex and ethnic group was investigated.^35^

Previous studies have indicated interaction between EBV and CMV infection on atopy/IgE sensitization,^18^, ^19^ so we compared the proportion of children who were atopic for combinations of age at CMV/EBV/VZV infection.

The number and type of positive allergens per child were described and examined by ethnic group and sex.

## 3 RESULTS

SPT was completed at the 4‐year visit for 740 children with serological data. Of these, 135 (18%) were atopic (95% CI 16‐21).

Table 1 shows the characteristics of the children and their mothers.

**Table 1:** Characteristics of the 740 children and their mothers and associations with atopy at age 4 y

	N (%)	Number of children atopic (%)	Unadjusted OR, 95% CI, *P*	Adjusted OR, 95% CI, *P*
Age at CMV infection				
By 12 mo	181 (24%)	31 (17)	1.02 (0.65‐1.60), 0.95	See Table 2 for sex‐specific estimates
12‐24 mo	74 (10%)	22 (30)	2.08 (1.20‐3.61), 0.009	
Uninfected at 24 mo	485 (66%)	82 (17)	1.0	
Age at EBV infection				
By 12 mo	112 (15%)	21 (19)	1.07 (0.63‐1.83), 0.80	See Table 2 for sex‐specific estimates
12‐24 mo	193 (26%)	37 (19)	1.10 (0.71‐1.70), 0.66	
Uninfected at 24 mo	435 (59%)	77 (18)	1.0	
Age at VZV infection				
By 12 mo	66 (9%)	12 (18)	1.00 (0.52‐1.95), 0.99	See Table 2 for sex‐specific estimates
12‐24 mo	128 (17%)	24 (19)	1.04 (0.64‐1.71), 0.87	
Uninfected at 24 mo	546 (74%)	99 (18)	1.0	
Mother's ethnic group				
White British	258 (35%)	28 (11%)	1.0	1.0
Pakistani	386 (52%)	89 (23%)	2.46 (1.56‐3.89), <0.001	1.78 (0.96‐3.31), 0.07
Other	96 (13%)	18 (19%)	1.90 (0.99‐3.61), 0.05	1.36 (0.65‐2.85), 0.41
Sex				
Female	343 (46%)	40 (12%)	1.0	1.0
Male	397 (54%)	95 (24%)	2.38 (1.59‐3.56), <0.001	3.28 (1.87‐5.76), <0.001^a^
Birth order				
1	257 (35%)	47 (18%)	1.0	1.0
2	208 (28%)	31 (15%)	0.78 (0.48‐1.28), 0.33	0.73 (0.43‐1.25), 0.25
3+	275 (37%)	57 (21%)	1.17 (0.76‐1.80), 0.48	0.86 (0.53‐1.41), 0.56
Duration of breastfeeding				
Never	126 (17%)	20 (16%)	1.0	1.0
0‐12 mo	510 (69%)	88 (17%)	1.11 (0.65‐1.88), 0.71	1.19 (0.66‐2.13), 0.56
>12 mo	102 (14%)	26 (25%)	1.81 (0.94‐3.48), 0.07	1.93 (0.93‐4.00), 0.08
Missing	2			
Regular childcare attendance by 24 mo				
Ever	293 (40%)	41 (14%)	0.61 (0.41‐0.91), 0.02	0.79 (0.49‐1.29), 0.35
Never	447	94 (21%)	1.0	1.0
Low birthweight (<2500 g)				
Yes	63 (9%)	16 (25%)	1.60 (0.88‐2.91), 0.13	1.90 (1.00‐3.62), 0.05
No	677	119 (18%)	1.0	1.0
Pets in the home (at 12 mo)				
Yes	185 (25%)	25 (14%)	0.63 (0.39‐1.01), 0.06	0.87 (0.50‐1.49), 0.60
No	550	109 (20%)	1.0	1.0
Missing	5			
Damp (damp spots on walls or ceilings) at 12 mo				
Yes	168 (23%)	42 (25%)	1.70 (1.12‐2.57), 0.01	1.68 (1.08‐2.62), 0.02
No	567	93 (16%)	1.0	1.0
Missing	5			
Smoking in pregnancy (asked at maternal baseline questionnaire)				
Yes	85 (12%)	9 (11%)	0.50 (0.24‐1.02), 0.06	0.72 (0.33‐1.57), 0.41
No	654	126 (19%)	1.0	1.0
Missing	1			
Central heating at 12 mo				
Yes	706 (96%)	126 (18%)	1.0	1.0
No	30	9 (30%)	1.97 (0.88‐4.41), 0.10	2.20 (0.93‐5.19), 0.07
Missing	4			
Maternal education				
5 GCSE equivalent or less	334 (45%)	67 (20%)	1.0	
A level equiv & higher	341 (46%)	60 (18%)	0.85 (0.58‐1.25), 0.41	
Other/DK/foreign unknown	64 (9%)	8 (13%)	0.57 (0.26‐1.25), 0.16	
Missing	1			
Home ownership				
Yes	470 (64%)	85 (18%)	1.0	
No	269	50 (19%)	1.03 (0.70‐1.52), 0.87	
Missing	1			
Maternal history of eczema, asthma or hay fever^b^				
Yes	284 (38%)	47 (17%)	0.83 (0.56‐1.22), 0.35	
No	456	88 (19%)	1.0	
Paternal history of eczema, asthma or hay fever^b^				
Yes	231 (31%)	45 (19%)	1.13 (0.76‐1.68), 0.56	
No	509	90 (18%)	1.0	
Term delivery (≥37 wk)				
Yes	696 (94%)	127 (18%)	1.00 (0.46‐2.21), 0.99	
No	44	8 (18%)	1.0	
Mode of delivery				
Vaginal	589 (80%)	106 (18%)	1.0	
Caesarean section	146	28 (19%)	1.08 (0.68‐1.72), 0.74	
Missing	5			

Note

Adjusted ORs only presented for variables included in the multiple logistic regression model for age at CMV infection.

a Adjusted OR shows main effect of sex (ie, among CMV uninfected) from the model including interaction term between sex and age at CMV infection.

b Similar results if limited to maternal/paternal history of eczema or hay fever (as not all asthma is atopic).

### 3.1 Unadjusted analysis

Among the children who were CMV infected by 12 months and those who remained uninfected at 24 months, 17% were atopic (31/181 and 82/485, respectively). However, of the 74 children who were CMV infected between 12 and 24 months, 30% (22) were atopic. There were no differences in the proportion of children atopic by age at EBV infection or by age at VZV infection (18% or 19% in each group).

Boys were twice as likely to be atopic than girls, and Pakistani children were twice as likely to be atopic than White British children. Childcare attendance by age 24 months, reporting pets in the home and central heating at the 12‐month visit, and smoking in pregnancy were associated with reduced risk of atopy. Low birthweight and damp reported at 12 months were associated with an increased risk of atopy (Table 1). There was not strong evidence of an association with pets and damp reported at the 24‐month visit. There was no evidence of associations between atopy and mould in the home, type of windows in child's bedroom, gas for cooking, gas fire, wood or coal fire with chimney, type of flooring or bedding (data not shown).

Only nine respondents reported making changes due to asthma/allergies (mainly changing to hard floors or new carpets, different bedding) at the 12‐month questionnaire (three were atopic), and only 17 reported making changes at the 24‐month questionnaire (five were atopic).

The associations of atopy with breastfeeding, childcare, pets and smoking in pregnancy may reflect differences in the prevalence of these factors between white British and Pakistani children; Pakistani women were more likely to breastfeed, less likely to use childcare and far less likely to smoke than white British women. Pakistani families were less likely to have pets than white British families.

The 740 children included here were generally similar to the total 1000 children with serological data, although there was a slightly higher proportion of Pakistani children.

### 3.2 Adjusted analysis

There was some evidence of effect modification of the association between age at CMV infection and atopy by sex (LRT χ^2^ = 4.80, *P* = 0.09), adjusting for ethnic group, birth order, duration of breastfeeding, regular childcare attendance by 24 months, low birthweight, pets in the home (12 months), damp (12 months), smoking pregnancy and central heating (12 months). In boys, there was no association between CMV infection in the second year and odds of atopy (adjusted OR [aOR] 1.20, 95% CI 0.52‐2.79) and weak evidence of a protective effect of infection before 12 months (aOR 0.57, 95% CI 0.31‐1.04). In girls, there was a strong association of CMV infection in the second year with increased odds of atopy (aOR 4.38, 95% CI 1.87‐10.29) and no association with infection in the first year (aOR 0.97, 95% CI 0.40‐2.33; Table 2).

**Table 2:** Sex‐specific estimates for the association between age at CMV, EBV and VZV infection and atopy

	n	No. atopic (%)	Unadjusted OR, 95% CI, *P*	Adjusted OR^a^, 95% CI, *P* n = 732
Age at CMV infection				
Girls				
By 12 mo	77	9 (12)	1.43 (0.62‐3.31), 0.41	0.97 (0.40‐2.33), 0.94
12‐24 mo	42	12 (29)	4.32 (1.90‐9.78), <0.001	4.38 (1.87‐10.29), 0.001
Uninfected at 24 mo	224	19 (8)	1.0	1.0
Boys				
By 12 mo	104	22 (21)	0.84 (0.49‐1.46), 0.54	0.57 (0.31‐1.04), 0.07
12‐24 mo	32	10 (31)	1.43 (0.64‐3.18), 0.38	1.20 (0.52‐2.79), 0.67
Uninfected at 24 mo	261	63 (24)	1.0	1.0
Age at EBV infection				
Girls				
By 12 mo	47	3 (6)	0.60 (0.17‐2.09), 0.42	0.42 (0.12‐1.53), 0.19
12‐24 mo	91	16 (18)	1.87 (0.92‐3.78), 0.08	1.64 (0.79‐3.39), 0.18
Uninfected at 24 mo	205	21 (10)	1.0	1.0
Boys				
By 12 mo	65	18 (28)	1.19 (0.64‐2.21), 0.58	0.99 (0.52‐1.90), 0.98
12‐24 mo	102	21 (21)	0.81 (0.46‐1.42), 0.45	0.72 (0.40‐1.32), 0.29
Uninfected at 24 mo	230	56 (24)	1.0	1.0
Age at VZV infection				
Girls				
By 12 mo	32	2 (6)	0.57 (0.13‐2.51), 0.45	0.52 (0.11‐2.42), 0.41
12‐24 mo	64	12 (19)	1.96 (0.93‐4.14), 0.08	2.85 (1.29‐6.30), 0.009
Uninfected at 24 mo	247	26 (11)	1.0	1.0
Boys				
By 12 mo	34	10 (29)	1.29 (0.59‐2.82), 0.52	1.60 (0.70‐3.67), 0.27
12‐24 mo	64	12 (19)	0.71 (0.36‐1.41), 0.33	0.79 (0.39‐1.61), 0.52
Uninfected at 24 mo	299	73 (24)	1.0	1.0

a Adjusted for ethnic group, birth order, duration of breastfeeding, regular childcare attendance by 24 mo, low birthweight, pets in the home (12 mo), damp (12 mo), smoking in pregnancy and central heating (12 mo).

There was also evidence of effect modification by sex of the association between age at EBV infection and atopy (LRT χ^2^ = 5.87, *P* = 0.05) and age at VZV infection and atopy (LRT χ^2^ = 8.52, *P* = 0.01); the sex‐specific estimates are presented in Table 2. Girls infected with VZV in the second year of life were at increased odds of atopy (aOR 2.85, 95% CI 1.29‐6.30). There was no evidence of effect modification by ethnic group of the association between age at CMV, EBV or VZV infection and atopy.

Table 1 shows the adjusted estimates (from the model for age at CMV infection) for the other variables associated with atopy. Boys had three times the odds of atopy overall compared to girls. The increased risk of atopy among Pakistani compared to White British children attenuated in multivariable analysis. There was weak evidence that children of low birthweight, those breastfed for more than 12 months and children living in homes without central heating had around twice the odds of atopy. There was good evidence that children living in damp homes were more likely to be atopic.

### 3.3 Infection with more than one virus in the second year is associated with greater risk of atopy

Of the total 740 children, 23 were infected with CMV and EBV between 12 and 24 months; 8 of 23 (35%) were atopic. Among 33 children who were CMV and EBV infected by 12 months, 6 (18%) were atopic. Similarly, among 121 children who were CMV and EBV infected by 24 months, 25 (21%) were atopic. Of 20 children who were CMV and VZV infected between 12 and 24 months, 9 (45%) were atopic, and of five children who were infected with all three viruses between 12 and 24 months, 4 (80%) were atopic. The final model for age at CMV infection, stratified by EBV infection status at 24 months, gave similar estimates to the overall results (data not shown).

### 3.4 Ethnic group differences in atopy

Among 386 Pakistani mothers, 250 were born outside, and 136 were born in, the UK. Sixty‐four (26%) children of those born outside the UK were atopic compared to 25 (18%) children of women born in the UK (χ^2^ = 2.59, *P* = 0.11). Twenty‐seven (11%) children of White British women born in the UK were atopic. Only two of the White British women were born abroad (and one child was atopic).

### 3.5 Which allergens were positive?

There were five allergens in the panel. Most of the 135 atopic children were positive for one (n = 56) or two (n = 52) allergens (Table 3). Positive reactions to house dust mites (HDM) were most common, followed by grass mix with positive cat and dog results the least common. The number of positive allergens per child did not vary substantially by ethnic group or sex.

**Table 3:** Number of positive reactions by allergen

	Number of positive reactions ≥ 3 mm				
	1	2	3	4	Total
Cat	1 (2%)	5 (5%)	5 (8%)	3 (12%)	
Dog	2 (4%)	0	2 (3%)	3 (13%)	
Grass mix	13 (23%)	6 (6%)	16 (25%)	6 (25%)	
Mite 1 (D. Pter)	27 (48%)	48 (46%)	20 (32%)	6 (25%)	
Mite 2 (D. Farin)	13 (23%)	45 (43%)	20 (32%)	6 (25%)	
	56	104 (52 children)	63 (21 children)	24 (6 children)	135

Pakistani children were more likely to have a positive test for HDM than White British children. Among 56 children who reacted to one allergen, 33 (83%) of 40 Pakistani children were positive to HDM compared to 5 (50%) of the 10 White British children (χ^2^ = 4.63, Fisher's exact *P* = 0.05). All except two children (1 WB, 1 Pakistani) with two positive reactions and all except one child (WB) with three positive reactions were positive for at least one HDM.

Among 56 children who reacted to one allergen, a higher proportion of boys were positive to HDM than girls but there was no evidence of a true difference (χ^2^ = 1.38, *P* = 0.24). Among 52 children who reacted to two allergens, all except one boy and one girl were positive for one or both HDM, and among 21 children with three positive reactions, all except one boy were positive for one or both HDM.

## 4 DISCUSSION

Our main finding is that, among the girls, CMV infection in the second year of life is associated with a fourfold increased odds of atopy compared to acquiring it in the first year or remaining uninfected at 2 years. Among the boys, there was no association between infection at 12‐24 months and atopy, but weak evidence of a protective effect of CMV infection in the first year of life on odds of atopy. Although age at EBV infection and age at VZV infection were not associated with risk of atopy in unadjusted analyses, there was good evidence of effect modification by sex. Stratified, adjusted estimates revealed an increased odds of atopy among girls infected with VZV in the second year of life compared to those still uninfected at 2 years.

Traditionally, until 50‐70 years ago in the UK and in low‐income countries today, most children became infected with CMV by the age of 12 months.^36^, ^37^ One explanation for our findings is that “delayed” infection, coinciding with immune development in the second year of life, may disrupt the balance of immune responses, leading to atopy. A second potential explanation is that CMV infection acquired when maternal antibody titres are declining might present a lower initial viral load to the immune system than found with perinatal infection from breast milk or infection later in life. Further studies are needed on this and should be linked to our observation of increased atopy in those of low birthweight who would have reduced transplacental passage of IgG.

The consistent direction of the sex‐specific estimates for CMV, EBV and VZV, with increased odds of atopy among girls infected in the second year with CMV and with VZV, supports the biological plausibility of a sex‐specific mechanism in the development of atopy. The “critical window” seems to be the second year of life when also considering combinations of CMV, EBV and VZV infections; the highest proportion of atopic children were among those infected in the second year of life with one or more viruses. Some evidence of a protective effect of CMV infection in the first year of life, at least in boys, fits with the traditional age at infection and corresponding low prevalence of atopy and demonstrates the importance of differentiating between infection in the first and second years.

Our results highlight potential sex‐specific effects of age at CMV infection on risk of atopy. Overall, boys had a greater risk of atopy than girls, as reported in other studies,^38^, ^39^ but differences in risk of atopy by age at CMV infection were only observed for girls. So why is “delayed” CMV infection associated with atopy in girls but not in boys?

There are other examples of sex differences in response or susceptibility to infections early in life,^40^, ^41^ and neonatal vitamin A supplementation (NVAS) was associated with increased atopy in girls but not boys in long‐term follow‐up of children enrolled in a previous randomized controlled trial in Guinea‐Bissau.^42^ As in our study, females were less likely to be atopic than males overall, but those receiving NVAS were at increased risk of atopy (adjusted RR = 1.78, 95% CI 1.17‐2.71) and wheeze during childhood (adjusted RR = 1.78, 95% CI 1.02‐3.09) compared to those receiving placebo. Possible mechanisms to explain these associations are that retinoic acids from vitamin A can lead to an enhanced Th2 response and a reduced Th1 response. High oestrogen levels can also promote a Th2 response, and girls experience surges in oestrogen levels in early infancy as well as in puberty. A further study by the group demonstrates that NVAS is associated with differences in response to BCG vaccination by sex and increased pro‐ to anti‐inflammatory cytokine ratios in females but decreased ratios in males, at 4‐6 months of age.^43^

In the Generation R cohort (population‐based cohort from foetal life onwards, in Rotterdam, the Netherlands), no interaction of sex and CMV infection was observed in relation to immune phenotypes but the children were tested at age 6 years,^35^ so differences by age at infection could not be investigated. In exploratory studies of the same cohort, an impact of female sex and CMV infection on immune cell dynamics in early childhood has been demonstrated.^44^

The combined evidence indicates that the sex‐specific associations we have observed may provide clues about the mechanisms involved in the development of atopy. There is evidence that CMV influences anti‐inflammatory mediators (viral and host IL‐10).^45^ CMV infection may therefore influence the Th1/Th2 balance particularly in the second year of life, and this, combined with hormonal effects in girls, could increase the risk of atopy. It is also possible that the reverse is true: early immune changes which lead to atopy may be associated with delayed CMV infection.

The group of children who were CMV infected in the second year is relatively small, only 10% of the total, and the majority of children in this cohort remained uninfected at age 2 years. Their risk of atopy was similar to those infected in the first year, and we do not know the age distribution of subsequent CMV infection in this group. However, we assume that CMV infection during early immune development, that is the first 2‐3 years, influences risk of atopy at age 4 years, and later infection has less impact.

We did not observe any differences in risk of atopy by age at EBV infection, in contrast to the Swedish studies,^19^ although there was evidence of effect modification by sex. EBV infection tends to be acquired later than CMV; in this cohort, most of the CMV infections occurred in the first year, usually through contact with the mother, whereas most EBV infections occurred in the second year via contact with siblings and other children.^26^ Delayed infection for EBV is therefore around age 3 or 4 years and older. A comparison of children infected by age 2 years (the “traditional” age at infection) with those infected between 2 and 4 years (delayed infection) would demonstrate any effect on risk of atopy, and we did not carry out serological testing at age 4 years. However, an effect on atopy risk would only be expected if the delayed infection coincided with a critical period of immune development. Similarly, VZV infection is usually acquired around age 3 or 4 years through contact with other children and we show that infection during the second year of life, a likely critical period of immune development, is associated with increased odds of atopy.

The 18% prevalence of atopy in our study is generally consistent with results from other European birth cohorts^19^, ^46^ and UK studies.^47^, ^48^ Some of these measured allergen‐specific IgE (sIgE) in serum rather than SPT, and the children included ranged from age 2 to 7 years with all primary school‐aged children included in one study.

The higher odds of atopy among Pakistani children, although attenuated in multivariable analysis, especially when including childcare in the model, is of interest, and there was some evidence of a higher risk of atopy in children of women born abroad and recently migrated to the UK. In the US NHANES study, allergic sensitization was associated with ethnicity and among those aged 1‐5 years was less prevalent in non‐Hispanic white children than other ethnic groups.^39^ The number of positive allergens per child did not vary by ethnicity, but there was weak evidence that Pakistani children were more likely to have a positive test to HDM than White British children. Higher levels of HDM and sensitization to HDM have been associated with damp in the home.^49^ In our study, a higher proportion of Pakistani children had damp in the home at 12 months than White British children, which might partly explain the higher odds of atopy in Pakistani children.

The key strength of our study is having infection status at both 12 and 24 months. To our knowledge, this is the first study to measure CMV‐ and EBV‐IgG at 12 and 24 months in relation to atopic status. The benefits of the cohort design included risk factor data collected at 12 and 24 months, and at 4 years, whereas cross‐sectional studies may not capture earlier exposures accurately either due to recall bias or if there are changes between age 1 and 4 years, which may have been made in response to allergic symptoms. There were few reported changes due to asthma or allergies at the 12‐ and 24‐month questionnaire.

SPT is widely used and accepted as a valid tool to define atopy.^28^ Several members of the team carried out the tests, and there may have been variations in technique between them, despite training and quality control checks. However, any measurement error and subsequent misclassification of atopic status would have been non‐differential, as the research team were unaware of the infection status of the children. Some studies use sIgE to define atopic status, instead of, or in addition to, SPT. There is generally good correlation between sIgE and SPT (>70% concordance), at least in high‐income country settings.^50^, ^51^

Not all children with a positive SPT will develop clinical symptoms of eczema, hay fever or allergic asthma. Some non‐atopic children may develop different types of asthma caused by non‐atopic mechanisms. Further work will investigate whether the higher odds of atopy among girls infected with CMV in the second year translates to an increased risk of eczema, hay fever, wheeze or asthma among these children.

In conclusion, our results highlight potential sex‐specific effects of age at CMV infection and age at VZV infection on risk of atopy, which may provide insight into the mechanisms involved in the development of atopy.

## CONFLICT OF INTEREST

The authors have no conflicts of interest in relation to this work.

## AUTHORS’ CONTRIBUTIONS

LP conceived and designed the study, analysed and interpreted the data and drafted and revised the manuscript. DW co‐ordinated data collection, interpreted the data and revised the manuscript. PG designed the study, interpreted the data and revised the manuscript. JW designed the study, interpreted the data and revised the manuscript. All authors have given final approval of the version to be published.
